# Timing of invasive strategy in patients with non-ST segment elevation acute myocardial infarction: A nationwide retrospective cohort analysis

**DOI:** 10.1007/s12471-025-01970-3

**Published:** 2025-08-04

**Authors:** Cyril Camaro, Marijke J. C. Timmermans, Judith A. van Erkelens, Chantal van Tilburg, Jan Reitsma, Dennis van Veghel, Karin E. Arkenbout, Peter Danse

**Affiliations:** 1 Department of Cardiology, Radboud University Medical Centre, Nijmegen, The Netherlands; 2 Netherlands Heart Registration, Utrecht, The Netherlands; 3 Dutch Business Intelligence Centre for Health Care (VEKTIS), Zeist, The Netherlands; 4 Dutch Association of Health Insurers (ZN), Zeist, The Netherlands; 5 Department of Cardiology, Tergooi Medical Centre, Hilversum, The Netherlands; 6 Department of Cardiology, Rijnstate Hospital, Arnhem, The Netherlands

**Keywords:** Non-ST segment elevation myocardial infarction, invasive coronary angiography, Percutaneous coronary intervention, National claims data, time to ICA, Time to PCI, length of stay, ACS guidelines

## Abstract

**Background:**

The Dutch ACS working group endorses a delayed invasive approach for non-ST segment elevation myocardial infarction (NSTEMI) patients as safe and acceptable. We analysed the timing of invasive coronary angiography (ICA) and percutaneous coronary intervention (PCI) for all patients admitted with NSTEMI.

**Methods:**

For this retrospective observational cohort study initiated by the Netherlands Heart Registration, we extracted Dutch medical claims and diagnosis codes for all NSTEMI patients who underwent PCI. Primary outcome was the time from hospital admission to ICA and PCI in patients admitted to PCI and non-PCI centres. Secondary analyses included the time from ICA to PCI and variation among individual PCI centres.

**Results:**

A total of 36,573 NSTEMI patients (median age 68 years, 30% female) were included in the analysis. 24,857 patients (68%) were admitted to a hospital with PCI facilities (*n* = 30) and 11,716 patients (32%) to a hospital without PCI on site (*n* = 42). ICA was performed < 3 days (72 h) in 33,476 patients (92%). For patients admitted in PCI centres ICA was performed < 3 days in 94% (*n* = 23,328), median 0 days (IQR 0–1) vs 87% (*n* = 10,148), median 1 day (IQR 1–2) in non-PCI centres. The longest delay (median 3 days; IQR 2–5) between ICA and PCI occurred in patients first admitted to non-PCI centres and transferred after local ICA.

**Conclusions:**

ICA within three days is achieved in a very high percentage of patients in both PCI and non-PCI centres. A clearly larger percentage receives PCI within three days when directly admitted to a PCI centre.

## What’s new?

- This retrospective observational cohort study represents a total of 36,573 patients with NSTEMI undergoing PCI between 2018 and 2021
- Invasive coronary angiography within three days is achieved in a very high percentage of patients in both PCI and non-PCI centres.
- A clearly larger percentage receives PCI within three days when directly admitted to a PCI centre.

## Introduction

The most recent 2023 guidelines for the management of acute coronary syndromes (ACS) recommend an early invasive strategy, defined as invasive coronary angiography (ICA) within 24 h of presentation, for high-risk patients with non-ST-segment elevation ACS (NSTE-ACS). This approach is supported by a class IIa recommendation with level A evidence for patients with established non-ST-segment elevation myocardial infarction (NSTEMI), dynamic ST- or T‑wave changes, transient ST-segment elevation, or a Global Registry of Acute Coronary Events (GRACE) risk score greater than 140 [1]. However, with regard to composite clinical endpoints, in 11 randomised clinical trials invasive coronary angiography and/or percutaneous coronary intervention (PCI) have no superiority over a delayed invasive strategy (< 72 h) [1]. There was, however an observed survival benefit with an early invasive strategy in patients with a GRACE risk score > 140 [2, 3]. Recognising variability in clinical practice, the Dutch ACS Working Group provided a schematic overview of key topics to address discrepancies in the management of high-risk NSTE-ACS patients in the Netherlands [4]. Their recommendations suggest that, for patients admitted directly to PCI-capable centres, ICA should ideally be performed within 24 h, but no later than 72 h in cases where logistical challenges arise. For patients initially admitted to non-PCI centres, transfer to a hospital with on-site PCI should be considered to ensure ICA is performed within 24 h, with a 72-hour window as a maximum permissible delay. A Dutch prospective single-centre registry demonstrated that only 59% of high-risk NSTE-ACS patients were treated in accordance with 2015 NSTE ACS guidelines [5]. Furthermore, a retrospective analysis of claims data from 9,641 patients covered by one of the largest health insurance companies in the Netherlands found that direct admission to PCI-capable centres for NSTE-ACS was associated with shorter hospital stays, faster time to revascularisation, fewer interhospital transfers, and reduced overall healthcare costs compared to patients initially presenting to non-PCI centres [6]. Data on adherence to the latest 2023 guidelines for NSTEMI patients are currently lacking. Therefore, we aim to evaluate the timing of ICA and PCI of NSTEMI patients treated with PCI in a nationwide retrospective cohort.

## Methods

This study is part of a quality project initiated by the Netherlands Heart Registration (NHR), in close collaboration with the Dutch Association of Health Insurers (Zorgverzekeraars Nederland) and the Dutch Health Care Information Centre (Vektis). The aim of the project was to investigate the feasibility of using claims data for quality improvement purposes and to create, without extra registration burden, relevant new insights for the monitoring and further improvement of the quality of care. A pilot project was initiated with the PCI registration committee of the NHR.

Within the NHR, cardiologists and cardiothoracic surgeons register baseline, procedural and outcome data for all invasive cardiac interventional, electrophysiological and surgical procedures. Through public reporting, the NHR supports cardiac patients, healthcare providers, and policymakers by making outcome data transparent [7]. The platform for the interpretation of the registered data is primarily embedded in registration committees, in which mandated cardiologists and cardiothoracic surgeons represent their hospitals. In cases of relevant outcome variation, healthcare delivery processes are discussed, and best practices are shared. In addition, quality improvement projects, including the presented study, are initiated. The PCI registration committee agreed to full transparency of the data in these quality projects.

## Study design

### National claims database

For this retrospective, observational cohort study, data were extracted from a national medical claims database (Vektis). Dutch residents (excluding military personnel and prisoners) who are (obliged) insured for health care costs are registered in a central database. These data consist of registered claims data of all health insurance companies in the Netherlands. All Dutch hospitals register diagnoses and procedures in the diagnosis-treatment combination (DBC), which is the current reimbursement system in the Netherlands. This system is comparable to the diagnosis-related group (DRG) system in other countries [8]. Eventually, the reimbursements are processed by insurance companies. These nationwide claims data are validated for quality assessments in the Netherlands. Baseline characteristics, diagnoses, and individual medication were highly accurate between the claims database and the patient records [9].

### Study population

The study population consisted of adult patients diagnosed with NSTEMI and treated with a PCI between 1 January 2018 and 31 December 2021. When multiple PCI’s were performed in the same patient within 365 days, we only included the first PCI in the analysis. As NSTEMI patients can be admitted to PCI centres as well as to non-PCI centres, both were included.

### Outcome measures

The primary outcome measures were the duration of hospitalisation prior to ICA and PCI. The length of stay was defined as the number of consecutive days between the date of the index admission and the date of the first ICA or PCI procedure. Length of stay was calculated for the total cohort, as well as for the separate years. Also the variation among individual centres was assessed.

The secondary outcome was the time from ICA to PCI, defined as the number of days between the index ICA and the date of the PCI procedure (acute PCI, one-vessel PCI, multivessel PCI, or PCI with FFR guidance). In cases where PCI was performed without a separately registered ICA, it ICA was assumed that ICA occurred simultaneously (in the same procedure) with the index PCI. The length of stay from ICA to PCI was obtained for all patients admitted to PCI and non-PCI centres. For patients admitted to non-PCI centres, the time interval between the date of local ICA and referral to a hospital with PCI facilities were also extracted.

### Statistical and data analysis

Continuous data were summarised as mean and standard deviation (SD) if normally distributed. In cases of non-normal distribution, medians with 25th and 75th percentiles were presented. Categorical data were summarised as frequencies and percentages. All analyses were performed for the total PCI group, and separately for patients primarily admitted to PCI centres and patients who were primarily admitted to a non-PCI centre. Data analyses were performed using SAS software version 9.4, SAS Institute Inc. (NC, United States).

## Results

A total of 36,573 patients with NSTEMI followed by PCI were included in the analysis. The median age was 68 years (IQR 59–76) and 30% of the cohort were female. A total of 24,857 patients (68%) were directly admitted to a hospital with PCI facilities on-site (*n* = 30), whereas 11,716 patients (32%) were initially sent to hospitals without PCI on-site PCI (*n* = 42). Baseline characteristics are presented in Tab. 1.

**Table 1:** Patient characteristics 2018–2021

	Total cohort (*n* = 36,573)	Patients admitted to PCI centre (*n* = 24,857)	Patients admitted to non-PCI centre (*n* = 11,716)
Age (years), median (IQR)	68 (59–76)	68 (58–76)	69 (60–77)
Female sex, *N* (%)	10,871 (30)	7,276 (29)	3,595 (31)

*IQR* interquartile range, *PCI* percutaneous coronary intervention

### Primary outcome measures

**Fig. 1:**
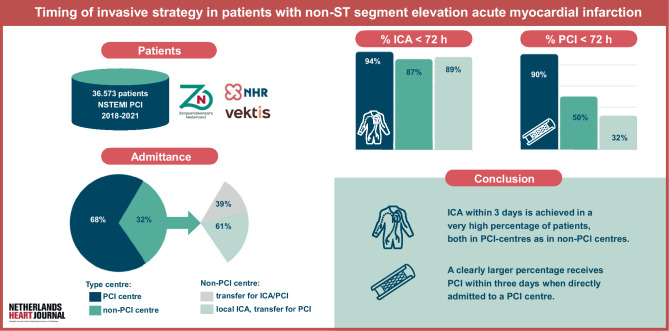
Infographic

**Fig. 2:**
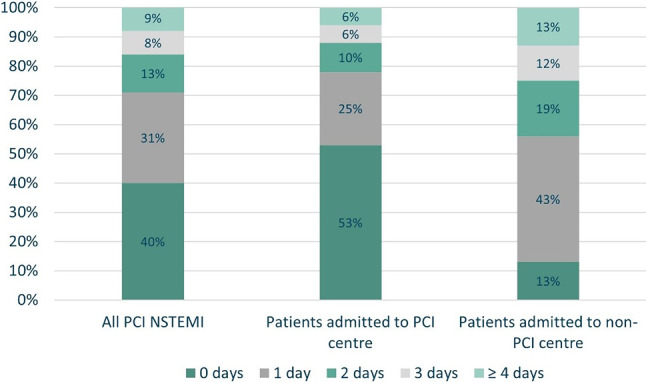
Time from admission to ICA. (*ICA* invasive coronary angiography, *NSTEMI* non-ST segment elevation myocardial infarction, *PCI* percutaneous coronary intervention)

For the total cohort, ICA was performed 3 days (< 72 h) in 33,476 patients (92%). The median time to ICA was 1 day (IQR 0–2). Among patients admitted to a PCI centre, ICA was performed within 3 days in 94% (*n* = 23,328), with a median time from admission of 0 days (IQR 0–1). In patients admitted to a non-PCI centre, ICA was performed within 3 days in 87% (*n* = 10,148), with a median time from admission of 1 day (IQR 1–2). (Infographic: Fig. 1). Figure 2 and Tab. 2 present the analysis of time to ICA.

**Table 2:** Timing of invasive strategy: PCI vs non-PCI centre (2018–2021)

	Total cohort (*n* = 36,573)	Patients admitted to PCI centre (*n* = 24,857)	Patients admitted to non-PCI centre (*n* = 11,716)	Patients admitted to non-PCI centre with local ICA (*n* = 7,124)
*Time from admission to ICA, median (IQR)*	1 (0–2)	0 (0–1)	1 (1–2)	1 (1–2)
– 0 days *n* (%)	14,726 (40.3%)	13,248 (53.3%)	1,478 (12.6%)	1,401 (19.7%)
– 1 days *n* (%)	11,159 (30.5%)	6,123 (24.6%)	5,036 (43.0%)	2,665 (37.4%)
– 2 days *n* (%)	4,844 (13.2%)	2,566 (10.3%)	2,278 (19.4%)	1,394 (19.6%)
– 3 days *n* (%)	2,747 (7.5%)	1,391 (5.6%)	1,356 (11.6%)	871 (12.2%)
– ≥ 4 days *n* (%)	3,097 (8.5%)	1,529 (6.2%)	1,568 (13.4%)	793 (11.1%)
*Time from ICA to PCI, median (IQR)*	0 (0–0)	0 (0–0)	1 (0–4)	3 (2–5)
– 0 days *n* (%)	27,784 (76.0%)	23,061 (92.8%)	4,723 (40.3%)	436 (6%)
– 1 days *n* (%)	1,537 (4.2%)	266 (1.1%)	1,271 (10.8%)	1,219 (17%)
– 2 days *n* (%)	1,708 (4.7%)	331 (1.3%)	1,377 (11.8%)	1,314 (18%)
– 3 days *n* (%)	1,307 (3.6%)	241 (1.0%)	1,066 (9.1%)	1,017 (14%)
– ≥ 4 days *n* (%)	4,237 (11.6%)	958 (3.9%)	3,279 (28.0%)	3,138 (44%)
*Time from admission to PCI, median (IQR)*	1 (0–3)	0 (0–2)	3 (2–6)	5 (3–7)
– 0 days *n* (%)	12,769 (34.9%)	12,769 (51.4%)	0 (0.0%)	0
– 1 days *n* (%)	8,447 (23.1%)	5,623 (22.6%)	2,824 (24.1%)	543 (7.6%)
– 2 days *n* (%)	4,165 (11.4%)	2,486 (10.0%)	1,679 (14.3%)	807 (11.3%)
– 3 days *n* (%)	2,828 (7.7%)	1,417 (5.7%)	1,411 (12.0%)	910 (12.8%)
– ≥ 4 days *n* (%)	8,364 (22.9%)	2,562 (10.3%)	5,802 (49.5%)	4,864 (68.3%)

*ICA* invasive coronary angiography, *IQR* interquartile range, *PCI* percutaneous coronary intervention

**Fig. 3:**
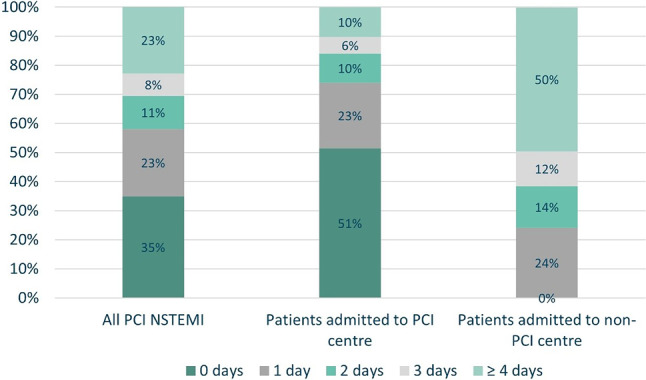
Time from admission to PCI. (*PCI* percutaneous coronary intervention, *NSTEMI* non-ST segment elevation myocardial infarction)

For the total cohort, 77% (*n* = 28,209) underwent PCI within 3 days. The median time to PCI was 1 day (IQR 0–3) and this was consistent throughout the years 2018–2021. PCI within 3 days was achieved in 90% of patients (*n* = 22,295) admitted to a PCI centre (median 0 days; IQR 0–2), compared to 50% of patients (*n* = 5,914) in a non-PCI centre (median 3 days; IQR 2–6). Figure 3 and Tab. 2 present the analysis of time to PCI.

### Secondary analyses

**Fig. 4:**
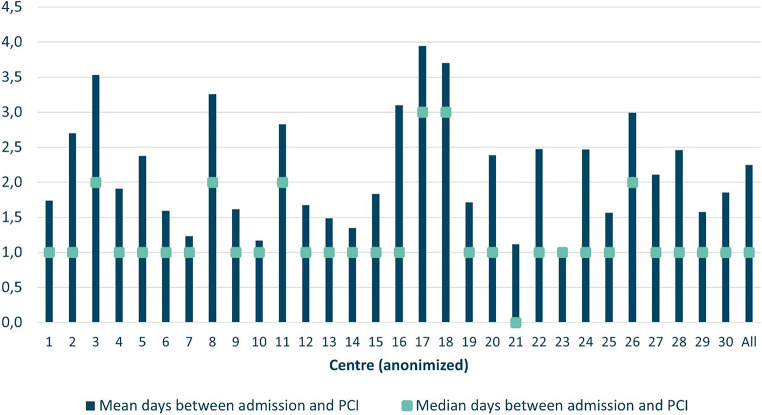
Time from admission to PCI, per individual PCI centre (2018–2021). (*PCI* percutaneous coronary intervention)

For the total cohort, median time form ICA to PCI was 0 days (IQR 0–0) as demonstrated in Tab. 2. The time window between ICA and PCI was median 0 days (IQR 0–0) in patients admitted in PCI-centres compared to 1 day (IQR 0–4) for the patients initially admitted in in non-PCI centres. 61% of patients (*n* = 7,124) admitted to a non-PCI centre underwent ICA before referral to a centre with PCI on-site. In these patients, ICA was performed within 3 days in 89% (*n* = 6,331) and PCI within 3 days in 32% (*n* = 2,260). The waiting time in these *n* = 7,124 patients between ICA and PCI was 3 days (IQR 2–5). Figure 4 illustrates the heterogeneity in waiting times for PCI across different centres.

## Discussion

The key finding of this nationwide retrospective cohort study was that in patients diagnosed with NSTEMI followed by PCI, an invasive evaluation (ICA) within 3 days (72 h) is achieved in a very high percentage of patients, both in PCI-centres (94%) as in non-PCI centres (87%). For the total cohort, 92% underwent ICA within 3 days, which is the time window as recommended by the Dutch ACS working group. However, when considering the latest European NSTE-ACS guidelines, the adherence rate of an early (< 24 h) invasive strategy in the Netherlands is below the class IIa recommendation of the ESC. Is should be noted that ICA within 24 h for stable NSTEMI patients (without very high-risk criteria) was downgraded from a class I to a class IIa recommendation, and therefore should be merely considered, as there is no superiority in death or non-fatal myocardial infarction in several large meta-analysis [1].

Overall adherence to the ESC guidelines showed conflicting results. In a national registry from Germany, university and academic hospitals showed the highest rate of guideline adherence, with almost 99% of the hospitals reporting immediate invasive management within 24 h in patients with NSTE-ACS [10]. Outside Europe, a nationwide registry study in China showed that hospitals had much less guideline adherence, with PCI performed within 72 h after admission in only 31% of patients with NSTE-ACS. One third of the patients with intermediate to very high risk did not undergo invasive evaluation or treatment at all [11].

Overall adherence to perform invasive coronary angiography meets the Dutch standards, although there is a variation in waiting time to PCI between the 30 Dutch PCI centres. The Dutch ACS Working Group endorses a delayed invasive strategy for NSTEMI patients, considering it both safe and acceptable [4]. Further reductions in waiting times could be achieved by sharing best practices (such as about in-hospital logistics, improvement of transfer and waiting times), which can encourage hospitals to shorten the time from diagnosis to ICA and subsequent PCI. Implementing best practices can further enhance the quality of care (i.e. patient satisfaction) for NSTEMI patients in the Netherlands.

Our secondary analysis showed that 19% of the total cohort (*n* = 7,124) underwent ICA in a non-PCI centre before being transported to a hospital with PCI facilities. Although guideline adherence is high even among patients who underwent local ICA in non-PCI centres (89%), this approach leads to a delay in PCI treatment. The longer waiting time in these patients is recognisable in real world. In a sub-analysis from the EARLY-ACS trial, timely angiography and revascularisation were often not achieved in transferred patients versus patients presented at hospitals with direct PCI capabilities [12]. However, it is still unknown if an early invasive strategy leads to better clinical outcomes [13, 14]. In a prospective multicentre two cohort study, the prehospital identification of patients eligible for direct transfer to PCI centres has proven feasible, leading to a reduction in hospital length of stay and healthcare costs [15]. Though transporting all NSTEMI patients to PCI centres would present significant logistical challenges for both ambulance services and catheterisation laboratories. Evidence from a prospective single-centre registry in the Netherlands indicates that only 59% of NSTEMI patients underwent PCI treatment [5]. In this registry, 23% received conservative treatment, while 18% required a CABG, suggesting a significant number of these transfers to PCI centres may have been unnecessary. Therefore, advice of a preferred admittance or a direct transfer of diagnosed NSTEMI patients to PCI centres remains a subject of ongoing debate.

Currently, no randomised clinical trials have evaluated the strategy of initial ICA at PCI versus non-PCI centres with respect to healthcare resources and costs. Reducing ambulance transports and the number of ICAs in non-PCI centres can enhance the efficiency of healthcare. Future studies have to address this issue.

### Strengths and limitations

An important strength of this study is the national coverage: national claim data of all health insurance companies in the Netherlands were analysed. Data from NSTEMI patients who underwent PCI were included. The validity of the use of medical claims data for quality purposes has been assessed by Van Eindhoven et al. by means of retrospective records reviews in patients with acute myocardial infarction. Baseline characteristics, diagnosis and individual medication were comparable between the national claims data and the medical records in four representative hospitals [9]. A limitation of this study is the inability to include patients’ clinical baseline characteristics, as this information was not available in the claims database. As a result, we were unable to identify NSTEMI patients who might benefit from early invasive coronary evaluation, such as those with high-risk criteria. In future, linking medical claims data with clinical data (e.g. from the Netherlands Heart Registration with Dutch Hospital Data) could provide valuable insights for identifying patients who would benefit most from early ICA and/or PCI [7]. Finally, time intervals were recorded in days, as the claims database did not provide time stamps with hour-level precision.

## Conclusion

In patients with NSTEMI treated with PCI, ICA within 3 days (72 h) is achieved in a very high percentage of patients, both in PCI-centres (94%) as in non-PCI centres (87%). A clearly larger percentage of patients receive PCI within three days when first admitted to a PCI centre.
